# Supplementary figures and images for: DNA double strand break repair in Escherichia coli perturbs cell division and chromosome dynamics
Source: PLoS Genet. 2020 Jan 2;16(1):e1008473. doi: 10.1371/journal.pgen.1008473 (PMC6959608; doi:10.1371/journal.pgen.1008473)

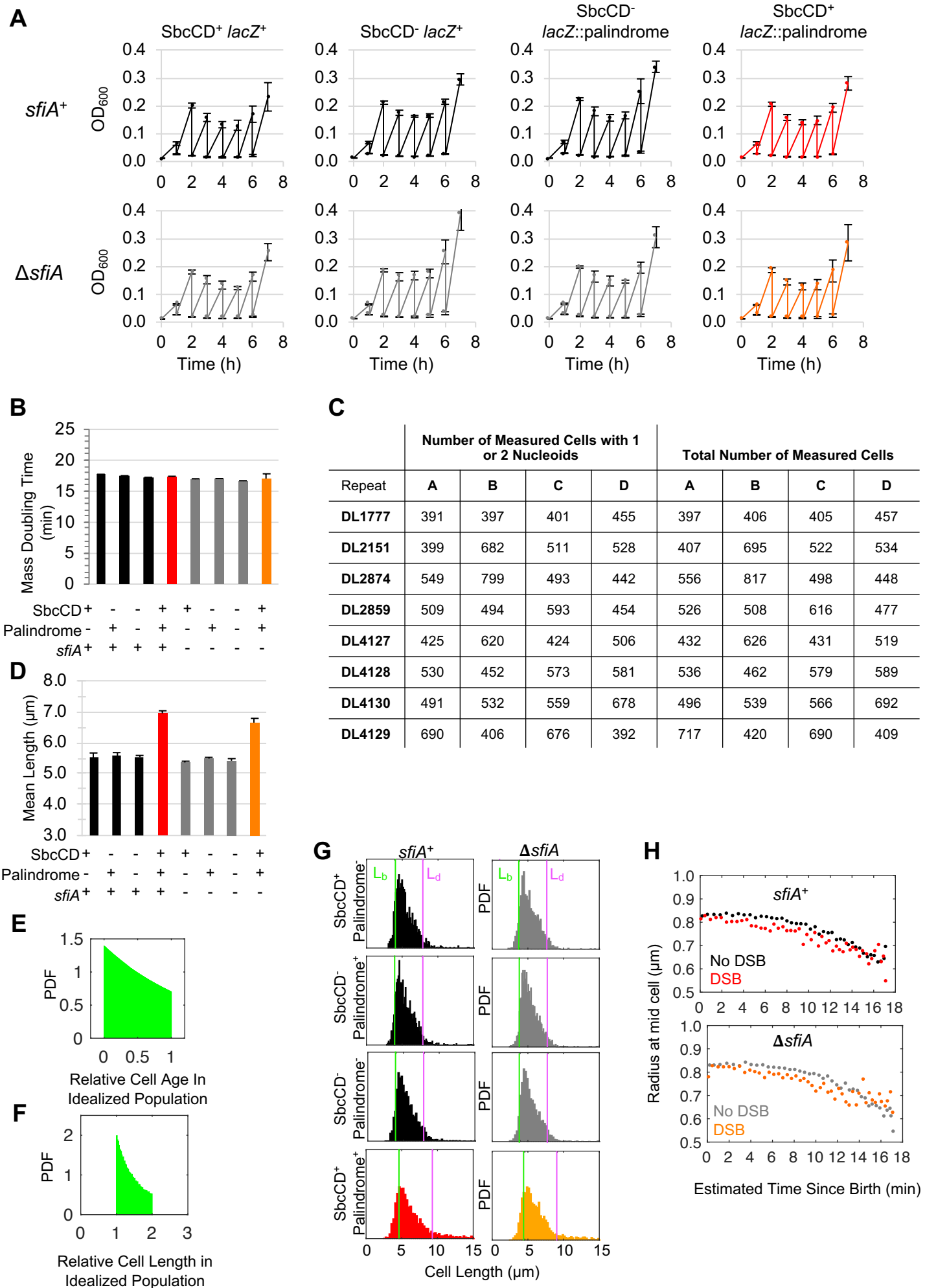

Supplement: S1 Fig — DSBR causes a SfiA-independent increase in average cell length at birth. A) Asynchronous cultures of E. coli were grown in rich growth media at 37°C and maintained in exponential growth phase by serial dilution. Population growth was measured as the optical density at 600nm (OD600). B) Average mass doubling time derived from rate of change of OD600. C) Number of cells analyzed following chloramphenicol-DAPI treatment. D) Mean cell length of cells containing either 1 or 2 chloramphenicol condensed DAPI stained nucleoids. For A, B and D, error bars show standard error of the mean, n = 4 independent cultures. E) Age structure of ideal asynchronous population with twice as many newborn cells as cells on the cusp of division. F) Expected distribution of relative cell lengths in an ideal asynchronous population assuming exponential growth. For E and F, data were normalized to approximate probability distribution functions (PDF). G) Histograms of measured cell lengths in asynchronous cultures with annotated estimated average length at birth (Lb) and estimated average length at division (Ld) calculated using the measured mean length of cells with either 1 or 2 chloramphenicol condensed nucleoids and assuming an ideal asynchronous population of cells. The data from four independent experiments for each E. coli strain were aggregated. H) Cell radius at mid-cell as a function of estimated time since birth, with estimated time since birth calculated as a conversion of measured cell length using the measured average mass doubling rate and estimated length at birth. For both sfiA+ and ΔsfiA strains, the data from the three control strain backgrounds not undergoing DSBR (SbcCD+ lacZ+, SbcCD- lacZ+ and SbcCD- lacZ::palindrome) were averaged for clarity in the plots. No difference was detected between the three control strains in either sfiA+ or ΔsfiA backgrounds. (PDF) [file pgen.1008473.s001.pdf]

A

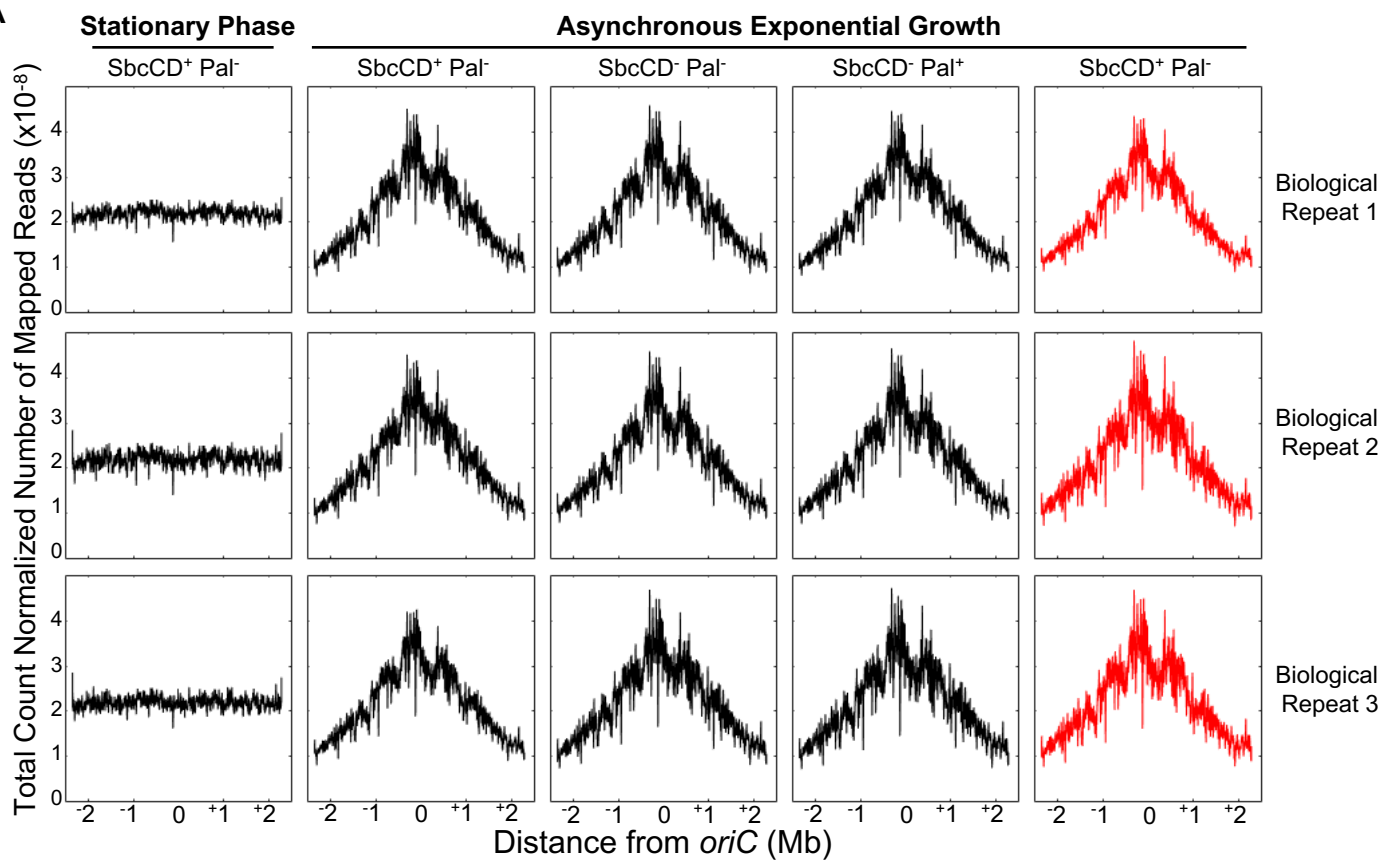

B

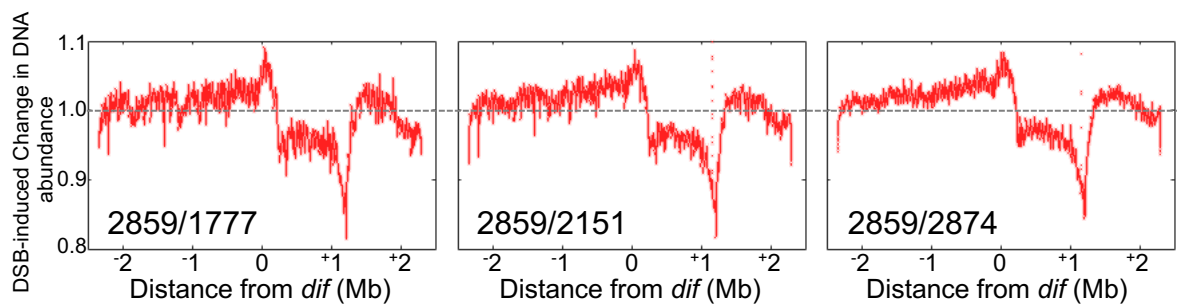

C

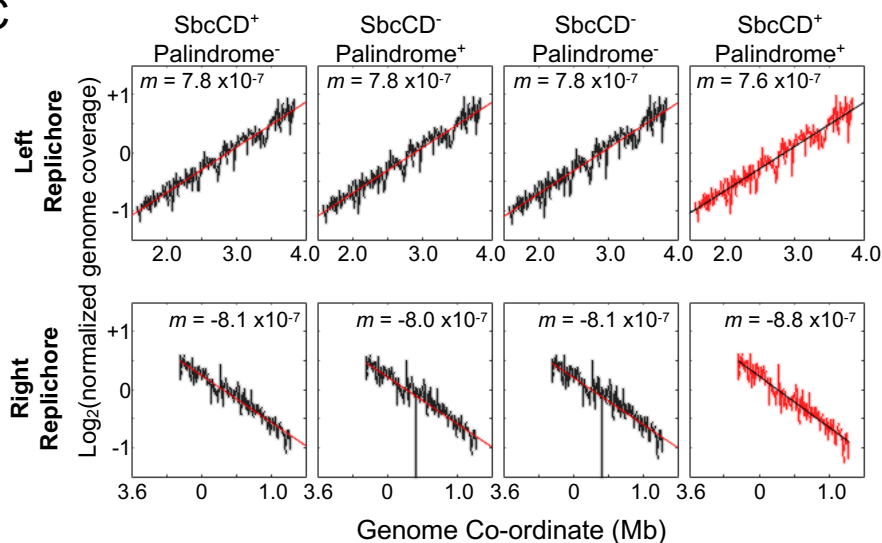

D

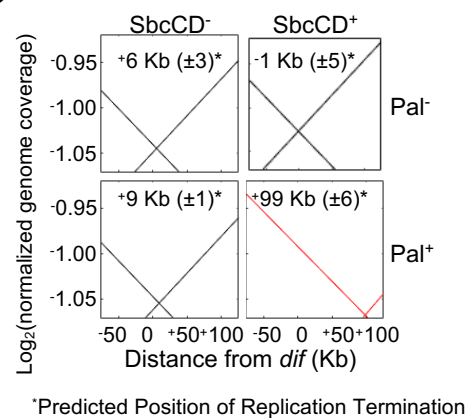

Supplement: S2 Fig — DSBR alters the chromosomal DNA replication profile without affecting the time required to complete DNA synthesis. A) The distribution of mapped sequencing reads across the genome for each of the biological repeats of the four strains. B) The mean MFA for three independent cultures of strain DL2859 (sbcCD+ lacZ::palindrome) experiencing DSBR at lacZ normalized against the mean MFA for three independent cultures of each of the three control strains (1777: sbcCD+ lacZ+; 2151: sbcCD- lacZ+; 2874: sbcCD- lacZ::palindrome). C) Linear regression of MFA results used to calculate the replication rates of the left and right replichores shown in Fig 3C. D) Intercept of the lines of best fit to the left and right replichores, used to calculate the predicted location of replication termination (Fig 3D). A moving mean of 10,000 bp was applied to the read counts to create the plots shown in A–C. (PDF) [file pgen.1008473.s002.pdf]

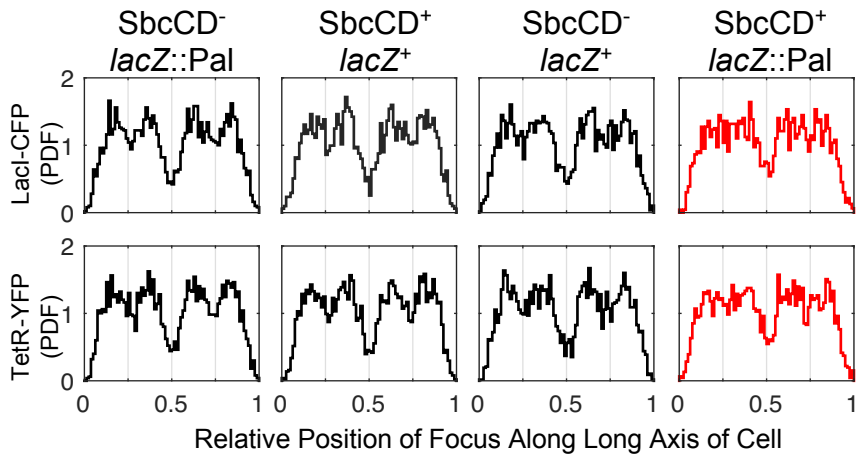

Supplement: S3 Fig — The locus undergoing DSBR dwells at future division planes. Spatial distribution of lacZ adjacent LacI-CFP and TetR-YFP foci along the long axis of cells undergoing DSBR at lacZ (SbcCD+ Palindrome+, red), or not. (PDF) [file pgen.1008473.s003.pdf]
