# Supplementary material for: DNA double strand break repair in Escherichia coli perturbs cell division and chromosome dynamics
Source: PLoS Genet. 2020 Jan 2;16(1):e1008473. doi: 10.1371/journal.pgen.1008473 (PMC6959608; doi:10.1371/journal.pgen.1008473)
Supplement: S1 Table — A list of E. coli strains used in this study. (PDF) [file pgen.1008473.s004.pdf]

Strain	Genotype	Figure	Source
MG1655	F⁻ λ⁻ *ilvG⁻ rfb-50 rph-1*		(Blattner, Plunkett et al. 1997)
BW27784	*lacI^q rrnB3* Δ*lacZ4787 hsdR514* Δ(*araBAD*)*567* Δ(*rhaBAD*)*568* Δ(araFGH) Φ (ΔParaE P_{CP18}-*araE*) *ydeV*::*IS1 valUX⁻*		(Khlebnikov, Datsenko et al. 2001)
DL1777	MG1655 *fnr-267 lacI^q lacZχ-*	Fig 1, 2, 3, 4, S1, S2	(Eykelenboom, Blackwood et al. 2008)
DL2151	MG1655 *fnr-267 lacI^q lacZχ-* Δ*sbcDC*	Fig 1, 2, 3, 4, S1, S2	(Eykelenboom, Blackwood et al. 2008)
DL2859	MG1655 *fnr-267 lacI^q lacZχ- lacZ*(L8) *lacZ*::*pal246 cynX*::Gm^R	Fig 1, 2, 3, 4, S1, S2	(Eykelenboom, Blackwood et al. 2008)
DL2874	MG1655 *fnr-267 lacI^q lacZχ- lacZ*(L8) Δ*sbcDC lacZ*::*pal246 cynX*::Gm^R	Fig 1, 2, 3, 4, S1, S2	(Eykelenboom, Blackwood et al. 2008)
DL4127	MG1655 *fnr-267 lacI^q lacZχ* Δ*sfiA*	Fig 1, 2, 4, S1,	(Darmon, Eykelenboom et al. 2014)
DL4128	MG1655 *fnr-267 lacI^q lacZχ-* Δ*sbcDC* Δ*sfiA*	Fig 1, 2, 4, S1,	(Darmon, Eykelenboom et al. 2014)
DL4129	MG1655 *fnr-267 lacI^q lacZχ- lacZ*::*pal246* Δ*sfiA*	Fig 1, 2, 4, S1,	(Darmon, Eykelenboom et al. 2014)
DL4130	MG1655 *fnr-267 lacI^q lacZχ-* Δ*sbcDC lacZ*::*pal246* Δ*sfiA*	Fig 1, 2, 4, S1,	(Darmon, Eykelenboom et al. 2014)
DL4695	BW27784 *rph⁺ ykgC*::P_{mw1}-*lacI-cerulean,tetR-Ypet hupA-mCherry* P_{BAD}-*sbcDC lacZ*::pal246 *cynX*::[(240x*tetO*)::Gm^R] *mhpC*::[(240x*lacO*)::Km^R] Δ*lacI lacZχ- mhpA*::χχχ *lacZ*::χχχ::*lacY*	Fig 5, S3	(White, Azeroglu et al. 2018)

DL4696	BW27784 *rph*$^+$ *ykgC*::P_{mw1}-*lacI-cerulean,tetR-Ypet hupA-mCherry* P_{BAD}-*sbcDC lacZ*$^+$ *cynX*::[(240x*tetO*)::Gm^R] *mhpC*::[(240x*lacO*)::Km^R] Δ*lacI lacZ*χ- *mhpA*::χχχ *lacZ*::χχχ::*lacY*	Fig 5, S3	(White, Azeroglu et al. 2018)

Gm^R, gentamycin resistance; Km^R, Kanamycin resistance; χ, Chi

References

Blattner, F. R., G. Plunkett, 3rd, C. A. Bloch, N. T. Perna, V. Burland, M. Riley, J. Collado-Vides, J. D. Glasner, C. K. Rode, G. F. Mayhew, J. Gregor, N. W. Davis, H. A. Kirkpatrick, M. A. Goeden, D. J. Rose, B. Mau and Y. Shao (1997). "The complete genome sequence of Escherichia coli K-12." Science **277**(5331): 1453-1462.

Darmon, E., J. K. Eykelenboom, M. A. Lopez-Vernaza, M. A. White and D. R. Leach (2014). "Repair on the Go: E. coli Maintains a High Proliferation Rate while Repairing a Chronic DNA Double-Strand Break." PloS one **9**(10): e110784.

Eykelenboom, J. K., J. K. Blackwood, E. Okely and D. R. Leach (2008). "SbcCD causes a double-strand break at a DNA palindrome in the Escherichia coli chromosome." Molecular cell **29**(5): 644-651.

Khlebnikov, A., K. A. Datsenko, T. Skaug, B. L. Wanner and J. D. Keasling (2001). "Homogeneous expression of the P(BAD) promoter in Escherichia coli by constitutive expression of the low-affinity high-capacity AraE transporter." Microbiology **147**(Pt 12): 3241-3247.

White, M. A., B. Azeroglu, M. A. Lopez-Vernaza, A. M. M. Hasan and D. R. F. Leach (2018). "RecBCD coordinates repair of two ends at a DNA double-strand break, preventing aberrant chromosome amplification." Nucleic Acids Res **46**(13): 6670-6682.
