# Supplementary material for: DNA double strand break repair in Escherichia coli perturbs cell division and chromosome dynamics
Source: PLoS Genet. 2020 Jan 2;16(1):e1008473. doi: 10.1371/journal.pgen.1008473 (PMC6959608; doi:10.1371/journal.pgen.1008473)
Supplement: S2 Table — A list of publicly accessible data generated in this study. (PDF) [file pgen.1008473.s005.pdf]

Sample	Source	Identifier
DL1777 Reference Sequence	This work; Fig 3, S2	GEO Series GSE141011
DL1777 Stationary Phase MFA Biological Repeat 1	This work; Fig 3, S2	GEO Series GSE141011
DL1777 Stationary Phase MFA Biological Repeat 2	This work; Fig 3, S2	GEO Series GSE141011
DL1777 Stationary Phase MFA Biological Repeat 3	This work; Fig 3, S2	GEO Series GSE141011
DL1777 Exponential Phase MFA Biological Repeat 1	This work; Fig 3, S2	GEO Series GSE141011
DL1777 Exponential Phase MFA Biological Repeat 2	This work; Fig 3, S2	GEO Series GSE141011
DL1777 Exponential Phase MFA Biological Repeat 3	This work; Fig 3, S2	GEO Series GSE141011
DL2151 Exponential Phase MFA Biological Repeat 1	This work; Fig 3, S2	GEO Series GSE141011
DL2151 Exponential Phase MFA Biological Repeat 2	This work; Fig 3, S2	GEO Series GSE141011
DL2151 Exponential Phase MFA Biological Repeat 3	This work; Fig 3, S2	GEO Series GSE141011
DL2859 Exponential Phase MFA Biological Repeat 1	This work; Fig 3, S2	GEO Series GSE141011
DL2859 Exponential Phase MFA Biological Repeat 2	This work; Fig 3, S2	GEO Series GSE141011
DL2859 Exponential Phase MFA Biological Repeat 3	This work; Fig 3, S2	GEO Series GSE141011
DL2874 Exponential Phase MFA Biological Repeat 1	This work; Fig 3, S2	GEO Series GSE141011
DL2874 Exponential Phase MFA Biological Repeat 2	This work; Fig 3, S2	GEO Series GSE141011
DL2874 Exponential Phase MFA Biological Repeat 3	This work; Fig 3, S2	GEO Series GSE141011
DL1777 Cell and Nucleoid Meshes (Oufti/MATLAB)	This Work; Fig 1, S1, 2, 4	10.6084/m9.figshare.9764975
DL2151 Cell and Nucleoid Meshes (Oufti/MATLAB)	This Work; Fig 1, S1, 2, 4	10.6084/m9.figshare.9764966
DL2859 Cell and Nucleoid Meshes (Oufti/MATLAB)	This Work; Fig 1, S1, 2, 4	10.6084/m9.figshare.9764984
DL2874 Cell and Nucleoid Meshes (Oufti/MATLAB)	This Work; Fig 1, S1, 2, 4	10.6084/m9.figshare.9764963
DL4127 Cell and Nucleoid Meshes (Oufti/MATLAB)	This Work; Fig 1, S1, 2, 4	10.6084/m9.figshare.9764978
DL4128 Cell and Nucleoid Meshes (Oufti/MATLAB)	This Work; Fig 1, S1, 2, 4	10.6084/m9.figshare.9764969
DL4129 Cell and Nucleoid Meshes (Oufti/MATLAB)	This Work; Fig 1, S1, 2, 4	10.6084/m9.figshare.9764981
DL4130 Cell and Nucleoid Meshes (Oufti/MATLAB)	This Work; Fig 1, S1, 2, 4	10.6084/m9.figshare.9764972
