# Supplementary material for: DNA double strand break repair in Escherichia coli perturbs cell division and chromosome dynamics
Source: PLoS Genet. 2020 Jan 2;16(1):e1008473. doi: 10.1371/journal.pgen.1008473 (PMC6959608; doi:10.1371/journal.pgen.1008473)
Supplement: S3 Table — A list of software/functions used and generated in this study. (PDF) [file pgen.1008473.s006.pdf]

Software/Function	Version	Source/Accession
getExtraDataLoop		This Work; https://github.com/mwhite4/ColiCellCycle_OuftiAnalyses
getCellLengths1or2Nucleoids		This Work; https://github.com/mwhite4/ColiCellCycle_OuftiAnalyses
getMidCellWidths		This Work; https://github.com/mwhite4/ColiCellCycle_OuftiAnalyses
avgCellCycleFromSnapshot_demograph		This Work; https://github.com/mwhite4/ColiCellCycle_OuftiAnalyses
Matplotlib	V2.0	matplotlib.org
fitderiv	v1.03	(Swain, Stevenson et al. 2016)
MetaMorph	v7	Molecular Devices
Autoquant	X2	Mediacy
MATLAB	vR2017a & vR2018a	Mathworks
OUFTI		(Paintdakhi, Parry et al. 2016)
FIJI	v2.0.0	(Schindelin, Arganda-Carreras et al. 2012)
Burrows-Wheeler Aligner (BWA-MEM)		http://bowtie-bio.sourceforge.net/bowtie2/index.shtml
SAMtools		(Li, Handsaker et al. 2009)

References

Li, H., B. Handsaker, A. Wysoker, T. Fennell, J. Ruan, N. Homer, G. Marth, G. Abecasis, R. Durbin and S. Genome Project Data Processing (2009). "The Sequence Alignment/Map format and SAMtools." Bioinformatics **25**(16): 2078-2079.

Paintdakhi, A., B. Parry, M. Campos, I. Irnov, J. Elf, I. Surovtsev and C. Jacobs-Wagner (2016). "Oufti: an integrated software package for high-accuracy, high-throughput quantitative microscopy analysis." Mol Microbiol **99**(4): 767-777.

Schindelin, J., I. Arganda-Carreras, E. Frise, V. Kaynig, M. Longair, T. Pietzsch, S. Preibisch, C. Rueden, S. Saalfeld, B. Schmid, J. Y. Tinevez, D. J. White, V. Hartenstein, K. Eliceiri, P. Tomancak and A. Cardona (2012). "Fiji: an open-source platform for biological-image analysis." Nat Methods **9**(7): 676-682.

Swain, P. S., K. Stevenson, A. Leary, L. F. Montano-Gutierrez, I. B. Clark, J. Vogel and T. Pilizota (2016). "Inferring time derivatives including cell growth rates using Gaussian processes." Nat Commun **7**: 13766.
